# Associations of depressive symptoms with white matter abnormalities and regional cerebral blood flow in patients with amnestic mild cognitive impairment

**DOI:** 10.1111/ggi.14467

**Published:** 2022-09-04

**Authors:** Kentaro Hirao, Fumio Yamashita, Hikaru Kato, Kyoko Kaneshiro, Akito Tsugawa, Rieko Haime, Raita Fukasawa, Tomohiko Sato, Hidekazu Kanetaka, Takahiko Umahara, Hirofumi Sakurai, Haruo Hanyu, Soichiro Shimizu

**Affiliations:** ^1^ Department of Geriatric Medicine Tokyo Medical University Tokyo Japan; ^2^ Division of Ultrahigh‐Field MRI, Institute for Biomedical Sciences Iwate Medical University Iwate Japan; ^3^ Present address: Ito clinic, 2‐12‐39 Shiranoe Moji‐ku, Kitakyushu Fukuoka Japan

**Keywords:** depressive symptoms, mild cognitive impairment, periventricular hyperintensities, regional cerebral blood flow, white matter hyperintensities

## Abstract

**Aim:**

Depressive symptoms are one of the most common neuropsychiatric symptoms in patients with mild cognitive impairment (MCI) and Alzheimer's disease (AD), although the pathophysiologies of the depressive symptoms that occur in these diseases have not been elucidated to date. In this study, we therefore investigated the associations between depressive symptoms and cognitive performance, white matter abnormalities, and regional cerebral blood flow (rCBF) in amnestic MCI patients.

**Methods:**

Thirty‐eight patients with amnestic MCI were analyzed. The volumes of periventricular hyperintensities (PVH) and deep white matter hyperintensities (DWMH) were measured on T2‐fluid‐attenuated inversion recovery magnetic resonance imaging using the imaging software 3D‐slicer. Associations between the Geriatric Depression Scale (GDS) score and other neuropsychological test scores on the one hand and the PVH and DWMH volumes on the other were analyzed. Voxel‐wise correlations of rCBF with GDS score, after controlling for the effects of age, were investigated using SPM8 software.

**Results:**

Significant correlations were identified between GDS score, Trail Making Test B and apathy scale scores on the one hand and PVH volume on the other. A significant negative association between GDS score and rCBF was identified in the right dominant bilateral dorsolateral prefrontal cortex (DLPFC).

**Conclusions:**

Depressive symptoms are significantly associated with PVH volume in MCI patients. The rCBF of the DLPFC was significantly associated with depressive symptoms, suggesting that this area might be closely involved in the pathogenesis of the depressive symptoms observed in MCI patients. **Geriatr Gerontol Int 2022; 22: 846–850**.

## Graphical abstract

Depressive symptoms are significantly associated with PVH, and the rCBF of the DLPFC was significantly associated with depressive symptoms, suggesting that the DLPFC might be closely involved in the pathogenesis of the depressive symptoms in MCI patients.

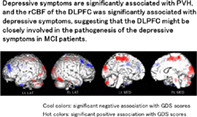

## Introduction

Depressive symptoms are one of the most common neuropsychiatric symptoms in patients with mild cognitive impairment (MCI) and Alzheimer's disease (AD), although the pathophysiology of the depressive symptoms observed in patients with these diseases has not been elucidated to date.^1^, ^2^ MCI with depressive symptoms has been reported to have a higher tendency of converting to dementia than MCI without depressive symptoms.^3^, ^4^ Furthermore, some studies have reported that white matter abnormalities are associated with depressive symptoms in MCI and AD,^5^, ^6^ and MCI patients with more white matter abnormalities show a higher risk of the onset of dementia.^7^ Although there have been several studies analyzing the association between regional cerebral blood flow (rCBF) and depressive symptoms in AD and MCI patients together,^8^, ^9^ to our knowledge few studies have analyzed the association of depressive symptoms with white matter hyperintensity (WMH) volume and rCBF only in amnestic MCI patients. In the present study, we therefore calculated the volume of periventricular hyperintensities (PVH) and deep white matter hyperintensities (DWMH) on T2‐fluid‐attenuated inversion recovery (FLAIR) magnetic resonance imaging (MRI) using the imaging software 3D‐slicer,^10^ and investigated the associations of the Geriatric Depression Scale (GDS)‐15 score with other neuropsychological scores, WMH volume, and rCBF.

## Materials and methods

### Subjects

Outpatients (aged > 60 years, < 90 years) who were enrolled at the memory clinic or outpatient clinic of Tokyo Medical University were prospectively recruited between 2015 and 2018. Written informed consent was obtained from all subjects before the study. The study design was approved by the ethics review board of Tokyo Medical University. Initial data of 38 subjects with amnestic MCI and of 10 normal control (NC) subjects, who were spouses of the MCI subjects or who were followed at the outpatient clinic but had no memory complaints, were medically stable, and had Mini Mental State Examination (MMSE) scores of 28 or above, were analyzed. All subjects underwent detailed general physical, neurological, and psychiatric examinations and extensive laboratory tests, including MRI and single‐photon emission computed tomography (SPECT). SPECT images were analyzed using Neurological Statistical Image Analysis software, which has three‐dimensional stereotactic surface projections developed by Minoshima *et al*. for evaluating the spatial distribution of abnormal perfusion to exclude other potential causes of dementia, including dementia with Lewy bodies, frontotemporal lobar degeneration, etc.^11^, ^12^ Reductions in rCBF of the parietotemporal association cortex on SPECT are recognized as a diagnostic pattern of prodromal AD.^13^, ^14^

MCI subjects were diagnosed as having MCI due to AD according to the National Institute on Aging–Alzheimer's Association criteria,^15^ and their MMSE scores were 24 or above. Subjects were excluded from the study if they had been prescribed any psychotropic drugs, including antidementia drugs and antidepressants, and they did not show any reduction in rCBF in the parietotemporal association areas. Subjects were also excluded if they had territorial or cortical infarctions, or if they showed severe white matter disease in which both PVH and DWMH were grade 3 on the Fazekas scale. Cognitive functions and neuropsychological symptoms, such as apathy and depressive symptoms, were assessed by various neuropsychological tests, such as the MMSE, Frontal Assessment Battery (FAB), Trail Making Test (TMT)‐A/B, Wechsler Memory Scale‐Revised‐Logical Memory I (immediate), verbal fluency (category), apathy scale, and GDS‐15.

Levels of serum cystatin C, 25‐hydroxyvitamin D, and homocysteine were measured using colloidal gold agglutination, radioimmunoassay, and high‐performance liquid chromatography, respectively. Other laboratory parameters for cerebrovascular risk factors, including total cholesterol, low‐density lipoprotein cholesterol, glucose, hemoglobin A1c, vitamin B12, blood urea nitrogen, creatinine, estimated glomerular filtration rate, aspartate transaminase, and alanine aminotransferase were also measured.

### Magnetic resonance imaging and volumetric analysis

Brain MRI scans (3D‐T1 and T2 FLAIR imaging) were performed using a 1.5‐tesla scanner (Magnetom; Siemens Medical Systems, Erlangen, Germany). FLAIR sequences were obtained with the following parameters: TR, 9000 ms; TE, 104 ms; TI, 2500 ms; slice thickness, 4.0 mm; and gap, 0.0 mm. For quantitative analysis of WMH volumes, PVH and DWMH lesions on FLAIR images were manually outlined by a neurologist using the 3D‐slicer software (http://www.slicer.org). Furthermore, intracranial volumes (ICVs) were calculated using the VBM toolbox, which was implemented in Statistical Parametric Mapping (SPM8, Wellcome Institute of Neurology, University College London, UK),^16^ and the ratio (%) of PVH and DWMH volumes to ICV was used for rating white matter abnormalities.

### SPECT imaging analysis

All analyses were performed using a triple‐head rotating gamma camera (PRISM 3000 XP, Picker) with a fan‐beam, permitting a spatial resolution of 6.8‐mm full width at half maximum, approximately 15 min after an intravenous bolus injection of 222 MBq of N‐isopropyl‐p‐(^123^I)‐iodoamphetamine. Prior to the injection, the subjects were allowed to rest in a quiet, dimly lit environment for 10 min. SPECT acquisition was undertaken in 24 steps (72 projections), in each of which counts were collected for 40 s. Reconstruction of the images was performed by filters (order: 8; cutoff: 0.40/cm) with attenuation correction (Chang method: 0.09/cm). The matrix size and slice thickness of the SPECT images were 128 × 128 and 4.3 mm, respectively.

Spatial preprocessing and the statistical analysis of images were performed on a voxel‐by‐voxel basis using SPM8. All SPECT images of each patient were normalized to the standard brain of the Montreal Neurological Institute, and spatial normalization was performed with 12‐parameter affine and nonlinear transformations. The voxel sizes of the reslice option were 2 mm × 2 mm × 2 mm. The nonlinear parameters were set at 25‐mm cut‐off basis functions and 16 iterations. All the normalized SPECT images were then smoothed with an isotropic Gaussian kernel filter (12‐mm full width at half maximum). To analyze the association between GDS score and rCBF in amnestic MCI patients, a simple regression method using SPM8 was applied. The analysis used thresholds of *P* < 0.01 and *P* < 0.001 (uncorrected) at the voxel level, and results were considered significant at 50 voxels at the cluster level. MNI coordinates of the peak voxels were converted to Talairach coordinates using Talairach Daemon (http://www.talairach.org/daemon.html), and anatomical regions were identified and presented using the Talairach coordinate system. To remove the effects of age, age was entered as a nuisance covariate.

### Statistical analysis

Demographic and laboratory data were calculated as means ± SD. Statistical analyses (Student *t*‐test, Mann–Whitney U‐test, Pearson's correlation coefficient, and Spearman's correlation coefficient) were performed using SPSS 26.0 software (IBM Corporation, Armonk, NY, USA). A *P*‐value of less than 0.05 was considered to be statistically significant.

## Results

**Figure 1:**
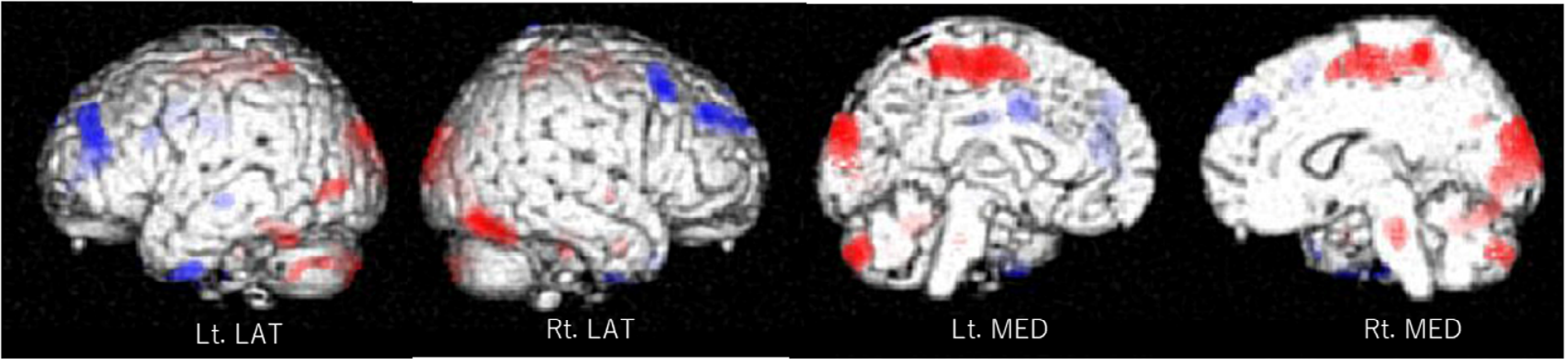
Association between rCBF and GDS‐15 scores. Cool colors indicate a significant negative association between the rCBF of the bilateral middle frontal gyrus, left inferior temporal gyrus, and bilateral anterior cingulate gyrus and GDS scores, and hot colors indicate a significant positive association between the rCBF of the bilateral paracentral gyrus, bilateral occipital lobe, and bilateral cerebellum and GDS scores, at a threshold of *P* < 0.01 (uncorrected), after controlling for the effects of age. rCBF, regional cerebral blood flow; Lt., left; Rt., right; LAT, lateral; MED, medial.

A summary of the comparison of the subjects' characteristics is shown in Table 1. We checked whether all items followed a normal distribution, and found that total WMH volume, PVH volume, and DWMH volume, and their ratios to ICV did not follow a normal distribution. Therefore, we used the Mann–Whitney U‐test for these items. There are significant differences between the MCI group and the NC group with regard to scores on neuropsychological tests, such as the MMSE, FAB, TMT‐B, and GDS‐15. On the other hand, although both the ratios of PVH and DWMH volumes to ICV in the MCI group are greater than in the NC group, the differences are not statistically significant. Table 2 shows that the GDS score is significantly correlated with TMT‐B and apathy scale scores by Pearson's correlation coefficient, and with the ratio of PVH volume to ICV by Spearman's correlation coefficient. In Figure 1, cool colors indicate that the rCBF of the bilateral middle frontal gyrus, left inferior temporal gyrus, and bilateral anterior cingulate gyrus is significantly negatively correlated with GDS scores, and hot colors indicate that the rCBF of the bilateral paracentral gyrus, bilateral occipital lobe, and bilateral cerebellum is significantly positively correlated with GDS scores, at a threshold of *P* < 0.01 (uncorrected), after controlling for the effects of age. Similar results were obtained without controlling for the effects of age (data not shown). Table 3 shows the probability results of the SPM analysis and the location of peak Z scores in terms of Talairach coordinates, at a threshold of *P* < 0.001 (uncorrected). There is a significant negative correlation of the rCBF of the right middle frontal gyrus in the dorsolateral prefrontal cortex (DLPFC) with GDS scores, whereas there is a significant positive correlation of the rCBF of the left medial frontal gyrus, left cerebellum, and right cuneus with GDS scores.

**Table 1:** Demographic, clinical, MRI and blood biochemistry data of the MCI and NC subjects

	MCI (*n* = 38)	NC (*n* = 10)	*P*‐value
Sex (M/F)	13/25	5/5	0.37
Age	77.4 ± 5.6	76.5 ± 6.2	0.49
Education (years)	13.4 ± 2.3	14.4 ± 1.6	0.07
MMSE	27.3 ± 1.6**	28.7 ± 0.9	0.001
FAB	13.0 ± 2.2*	14.9 ± 2.0	0.01
TMT‐A (s)	52.7 ± 20.8	42.2 ± 12.6	0.14
TMT‐B (s)	156.9 ± 80.8*	102.5 ± 39.6	0.05
WMS‐R‐logical memory (immediate)	13.6 ± 6.7	15.5 ± 5.9	0.13
VF (category)	14.5 ± 3.4	15.9 ± 3.4	0.26
GDS‐15	3.8 ± 3.1*	1.7 ± 1.4	0.05
Apathy scale	12.4 ± 4.7	12.4 ± 4.2	0.98
Total WMH vol. (mm^3^)	13 615 ± 14 725	8973 ± 10 792	0.2
PVH vol. (mm^3^)	9007 ± 7755	6172 ± 6255	0.22
DWMH vol. (mm^3^)	4608 ± 9334	2800 ± 5143	0.24
Total WMH vol. ratio to ICV (%)	1.04 ± 1.09	0.66 ± 0.79	0.18
PVH vol. ratio to ICV (%)	0.70 ± 0.60	0.45 ± 0.46	0.19
DWMH vol. ratio to ICV (%)	0.34 ± 0.69	0.20 ± 0.37	0.2
HbA1c (%)	5.9 ± 0.4	6.1 ± 0.5	0.14
T‐Cho (mg/dL)	205.0 ± 33.6	187.2 ± 17.6	0.11
LDL‐Cho (mg/dL)	113.1 ± 26.0	103.4 ± 17.6	0.28
eGFR (mL/min/1.73 m^2^)	66.2 ± 13.4	67.5 ± 18.9	0.8
Cystatin C (mg/L)	1.0 ± 0.2	1.1 ± 0.3	0.55
25(OH)VitD (ng/mL)	25.0 ± 11.9	19.9 ± 6.1	0.36
Homocysteine (nmoL/mL)	10.5 ± 3.5	9.4 ± 2.8	0.32
Systolic blood pressure (mmHg)	133.7 ± 17.4	132.2 ± 13.9	0.81
Diastolic blood pressure (mmHg)	73.7 ± 13.6	74.8 ± 11.1	0.82
Hypertension, n (%)	21 (55)	2 (20)	0.17
Diabetes mellitus, n (%)	7 (18)	3 (30)	0.43
Dyslipidemia, n (%)	20 (53)	4 (40)	0.49
Coronary artery disease, n (%)	3 (8)	1 (10)	0.84

Abbreviations: 25(OH)VitD, 25‐hydroxyvitamin D; eGFR, estimated glomerular filtration rate; FAB, Frontal Assessment Battery; GDS, geriatric depression scale; HbA1c, hemoglobin A1c; LDL‐Cho, low‐density lipoprotein cholesterol; MCI, mild cognitive impairment; MMSE, Mini Mental State Examination; NC, normal control; T‐Cho, total cholesterol; TMT, Trail Making Test; VF, verbal fluency; WMS‐R, Wechsler memory scale‐revised.

* *P* < 0.05 between MCI and NC.

^**^ *P* < 0.005 between MCI and NC.

**Table 2:** Correlations of GDS‐15 scores with age, other neuropsychological test scores, and WMH volume ratio to ICV in amnestic MCI patients

	Coefficient	*P*‐value
Age	0.07	0.68
MMSE	0.16	0.34
FAB	−0.24	0.15
TMT‐A (s)	0.32	0.06
TMT‐B (s)	0.37*	0.03
WMS‐R‐logical memory (immediate)	−0.09	0.58
VF (category)	−0.26	0.12
Apathy scale	0.43**	0.009
Total WMH vol. ratio to ICV (%)	0.25	0.08
PVH vol. ratio to ICV (%)	0.3*	0.04
DWMH vol. ratio to ICV (%)	0.11	0.44

Abbreviations: DWMH, deep white matter hyperintensities; FAB, Frontal Assessment Battery; GDS, Geriatric Depression Scale; ICV, intracranial volume; MCI, mild cognitive impairment; MMSE, Mini Mental State Examination; PVH, periventricular hyperintensities; TMT, Trail Making Test; VF, verbal fluency; WMH, white matter hyperintensities; WMS‐R, Wechsler memory scale‐revised.

* *P* < 0.05, significant correlation with GDS‐15 score.

^**^ *P* < 0.01, significant correlation with GDS‐15 score.

**Table 3:** Brain regions significantly associated with GDS‐15 scores in amnestic MCI patients

	Number of voxels	Peak Z scores	Coordinates (Talairach)			Structure
			x	y	z	
Regions with a significant positive correlation between rCBF and GDS scores	318	3.74	−7	−24	54	Left medial frontal gyrus
	144	3.56	−3	−78	−39	Left uvula of cerebellum
	190	3.56	4	–87	12	Right cuneus
Regions with a significant negative correlation between rCBF and GDS scores	102	4.07	41	12	53	Right middle frontal gyrus

Results are shown at a threshold of *P* < 0.001 (uncorrected).

Abbreviations: GDS, Geriatric Depression Scale; MCI, mild cognitive impairment; rCBF, regional cerebral blood flow.

## Discussion

In this study, we found that there were significant differences in GDS scores between the MCI group and the NC group. Although the severity of the depressive symptoms was mild, the MCI group showed more depressive symptoms than did the NC group, which is consistent with the results of previous reports.^1^, ^2^ Furthermore, we found that the GDS score was significantly correlated with the TMT‐B and apathy scale scores, and with the ratio of PVH volume to ICV. The association of depressive symptoms with executive dysfunction is consistent with the results of previous studies on AD patients.^17^, ^18^ Although depressive symptoms have been reported to be associated with white matter abnormalities,^5^, ^6^ in the present study only the ratio of PVH volume to ICV was correlated with depressive symptoms. We suspect that there might be a threshold for the DWMH volume that causes depressive symptoms in amnestic MCI patients, and we believe that the association between regional WMH volume and depressive symptoms should be investigated in the future. On the other hand, possible reasons why the ratio of DWMH volume to ICV was not correlated with depressive symptoms in this study are that subjects with severe WMH were not included, and hence the effects of WMH might not have been sufficiently detected, and also that the sample size was small, and hence the statistical power might have been insufficient.

We found that there were significant negative correlations between GDS scores and rCBF in the bilateral DLPFC, after controlling for the effects of age. These results are consistent with previous studies on AD patients.^8^, ^9^ Although it is difficult to interpret the laterality of the right or left dominance of the decreased rCBF in the DLPFC, we suspect that coexisting apathy and depressive symptoms might lead to right dominance of the decreased rCBF in the DLPFC, because the GDS test includes apathy symptoms, and GDS scores were significantly positively correlated with apathy scale scores in the present study, and apathy symptoms in AD patients have been reported to be associated with the right dominance of the decreased rCBF in the DLPFC.^8^, ^19^ Neuropathological and neuroimaging studies have consistently demonstrated the disruption of monoamine systems, particularly of the serotonin system, in MCI and AD patients.^20^, ^21^ Serotonin degeneration has been reported to be associated with depressive symptoms in AD. Correlation analyses have shown that glucose metabolism in the right DLPFC is positively correlated with the level of striatal serotonin transporters, suggesting that subcortical serotonergic dysfunction may affect cortical function in regions implicated in affective processing, such as in the DLPFC.^22^, ^23^ Some studies have reported that AD neuropathologies, such as amyloid‐β and neurofibrillary tangles, are associated with depression in cognitively normal subjects, as well as in MCI and AD patients.^24^, ^25^, ^26^, ^27^ Furthermore, neuroinflammation, which is associated with AD neuropathologies and white matter abnormalities, has been suggested to be associated with depressive symptoms.^28^, ^29^, ^30^ On the other hand, we found that there were positive significant associations between GDS scores and rCBF in the bilateral paracentral gyrus, bilateral occipital lobe, and bilateral cerebrum. We assume that the increased rCBF in the above regions might result from a compensatory reaction to affective processing. Considering the above, we believe that the DLPFC is significantly involved in the depressive symptoms caused by some AD‐associated pathophysiologies, including white matter abnormalities, because the MCI subjects were not prescribed any antidementia drugs, such as cholinesterase inhibitors or antidepressants. However, this does not mean that these associations are specific to amnestic MCI patients, because comparisons with other diagnostic groups were not performed in the present study. A better understanding of the underlying mechanisms of depressive symptoms in amnestic MCI patients will be important to establish appropriate treatment strategies for the prevention of the conversion from MCI to dementia, as well as for the prevention of depressive symptoms.

This study has some limitations. First, the sample size was small. However, it is clear from the results that the rCBF of the DLPFC is significantly associated with depressive symptoms in MCI patients. Second, as we excluded subjects with severe white matter diseases, our results should be interpreted with caution. Third, we did not apply corrections of brain atrophy for the SPECT data. Therefore, rCBF might be overestimated or underestimated by the partial volume effect (PVE). In particular, rCBF might be underestimated in atrophic regions. As the PVE might have affected the results of the association between rCBF and depressive symptoms in the present study, again, caution should be taken in interpreting the results. Fourth, the association between regional WMH volume and depressive symptoms was not assessed in this study, and we plan to investigate this association in the near future. Fifth, although the underlying pathology in MCI patients was not confirmed in this study, neuroimaging data were used as part of the diagnostic process. In particular, a decrease in rCBF in the parietotemporal association cortex on SPECT is recognized as a diagnostic pattern of MCI due to AD.^14^ Therefore, we are confident that most MCI patients did indeed have AD pathology.

In conclusion, PVH is significantly associated with depressive symptoms, and the rCBF of the DLPFC is significantly associated with depressive symptoms, suggesting that the DLPFC is closely involved in the pathogenesis of the depressive symptoms observed in amnestic MCI patients.

## Conflicts of interest disclosure

The authors have no conflicts of interest to declare regarding this study.

## Data Availability

The data that support the findings of this study are available from the corresponding author upon reasonable request.
